# Complete chloroplast genome sequence of *Fagopyrum dibotrys*: genome features, comparative analysis and phylogenetic relationships

**DOI:** 10.1038/s41598-018-30398-6

**Published:** 2018-08-17

**Authors:** Xumei Wang, Tao Zhou, Guoqing Bai, Yuemei Zhao

**Affiliations:** 1 School of Pharmacy, Xi’an Jiaotong University, Xi’an, 710061 China; 2 Shaanxi Engineering Research Centre for Conservation and Utilization of Botanical Resources, Xi’an Botanical Garden of Shaanxi Province, Xi’an, 710061 China; 3 College of Biopharmaceutical and Food Engineering, Shangluo University, Shangluo, 726000 China

## Abstract

*Fagopyrum dibotrys*, belongs to Polygonaceae family, is one of national key conserved wild plants of China with important medicinal and economic values. Here, the complete chloroplast (cp) genome sequence of *F. dibotrys* is reported. The cp genome size is 159,919 bp with a typical quadripartite structure and consisting of a pair of inverted repeat regions (30,738 bp) separated by large single copy region (85,134 bp) and small single copy region (13,309 bp). Sequencing analyses indicated that the cp genome encodes 131 genes, including 80 protein-coding genes, 28 tRNA genes and 4 rRNA genes. The genome structure, gene order and codon usage are typical of angiosperm cp genomes. We also identified 48 simple sequence repeats (SSR) loci, fewer of them are distributed in the protein-coding sequences compared to the noncoding regions. Comparison of *F. dibotrys* cp genome to other Polygonaceae cp genomes indicated the inverted repeats (IRs) and coding regions were more conserved than single copy and noncoding regions, and several variation hotspots were detected. Coding gene sequence divergence analyses indicated that five genes (*ndhK, petL rpoC2, ycf1, ycf2*) were subject to positive selection. Phylogenetic analysis among 42 species based on cp genomes and 50 protein-coding genes indicated a close relationship between *F. dibotrys* and *F. tataricum*. In summary, the complete cp genome sequence of *F. dibotrys* reported in this study will provide useful plastid genomic resources for population genetics and pave the way for resolving phylogenetic relationships of order Caryophyllales.

## Introduction

The angiosperm chloroplast (cp) genome is more conserved than the nuclear and mitochondrial genome; typically its structure is quadripartite, containing a pair of inverted repeats (IRs), a large single-copy (LSC) region, and a small single-copy (SSC) region^1^. The cp genomes of plants are highly conserved in gene structure, organization, and content^2^. Because of its conserved and non-recombinant nature, cp genomes are used as a robust tool in genomics and evolutionary studies^3^. And some evolutionary hotspots of plant plastid genome such as single nucleotide polymorphisms and insertion/deletions can provide useful information to elucidate the phylogenetic relationships of taxonomically unresolved plant taxa^4,5^.

Traditionally, chloroplasts were firstly isolated by means of sucrose gradient centrifugation. And then pure cpDNA extracted from chloroplasts was used for cp genome sequencing. This approach often resulted in high quality cpDNA, but requires enough fresh leaf materials (20~100 g) and special high-speed refrigerated centrifuge^6^. Combined with high costs of traditional Sanger sequencing, only a small portion of the cp genomes were obtained, which are insufficient for determining evolutionary relationships and applying on plant phylogenetic and genomic studies. Recently, with the advent of next generation sequencing (NGS), the cost of DNA sequencing was dramatically decreased and numbers of genome sequences were generated. Therefore, it is comparatively simple to obtain chloroplast genome sequences for plant species by using NGS than by traditional Sanger sequencing. Nowadays, hundreds of flowering plant cp genomes were sequenced by NGS technology and were applied to phylogenetic analyses at different taxonomical levels^7–9^.

The 27 species in the genus *Fagopyrum* (Polygonaceae) are commonly called ‘buckwheat’^10^. *Fagopyrum* is primarily distributed in Eurasia, especially in southwest of China. *Fagopyrum dibotrys* (D. Don) Hara. is a perennial herb with important medicinal and economic values. *Fagopyrum cymosum* (Trev.) Meisn. was once commonly treated as the synonym of *F. dibotrys*, as there is no description in Latin when *F. cymosum* was firstly published^11,12^. The dried rhizomes of *F. dibotrys* (*jin qiao mai*) is one of the famous traditional Chinese medicines for the treatment of lung disease, dysentery, rheumatism, throat inflammation, and the grains of *F. dibotrys* have high nutritional value and health benefits^13–16^. *Fagopyrum dibotrys* was once widely distributed in China and was an important ecological and genetic resource^17^. The wild resource of *F. dibotrys* has declined dramatically, however, due to overexploitation, few natural populations remain. So far, *F. dibotrys* has been designated as a national key conserved wild plant of China by the State Council of Traditional Chinese Medicine and listed in the *National Important Wild Conservation Plants in China*^13^.

Because of the nutritional and medicinal value of *F. dibotrys*, research has mainly focused on its pharmaceutically active components. There is little data concerning its genetic diversity based on genetic markers (e.g. allozyme)^18^, and the phylogenetic position of *F. dibotrys* was inferred using few genetic markers (e.g. RAPD, ITS, *rbcL* and *accD*) only^19–21^. *F. dibotrys* was once considered as the wild ancestor of common and Tartary buckwheat. But molecular studies indicated that *F. dibotrys* is closer to Tartary buckwheat than to common buckwheat and *F. dibotrys* is not the ancestor of cultivated buckwheat^20–22^. Therefore, more genetic markers are needed to clarify its still debatable phylogenetic positon^17^. Although complete cp genome sequences of some *Fagopyrum* species are now available^23–25^, a comprehensive phylogenetic analysis based on whole cp genomes has not been published. Thus, the availability of complete cp genomes that include new variable and informative sites should help to elucidate a more accurate phylogeny.

In this study, we obtained the complete cp genome of *F. dibotrys* based on Illumina paired-end sequencing followed by a *de novo* and reference guided assembly. We analyzed the genome features of *F. dibotrys* and compared them with cp genomes from Polygonaceae species. We performed a phylogenomic analysis using cp genomes and 50 shared cp genes to reconstruct the phylogeny of order Caryophyllales and infer the preliminary phylogenetic position of *F. dibotrys*.

## Results

### Genome assembly and genome features of *F. dibotrys*

**Figure 1:**
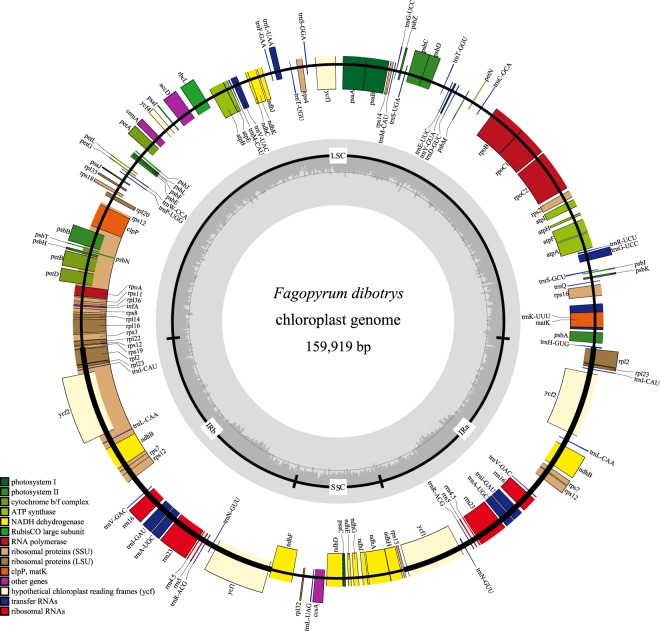
Chloroplast genome map of *F. dibotrys*. The genes drawn outside of the circle are transcribed counterclockwise, while those inside are clockwise. Small single copy (SSC), large single copy (LSC), and inverted repeats (IRa, IRb) are indicated. GC content is shown. Gene function or identifiers are displayed using colors indicated by the inner legend.

After Illumina paired-end sequencing, 24,970,664 reads were recovered with a sequence length of 125 bp. The total length of the reads was approximately 7.38 Gb and 24,959,432 clean reads were collected to assemble the *F. dibotrys* cp genome. Based on a combination of *de novo* and reference guided assembly, the cp genome of *F. dibotrys* was obtained. The complete cp genome of *F. dibotrys* was 159,919 bp in length and contained a pair of IRs (30,738 bp) which were separated by a small single copy (SSC) region (13,309 bp) and a large single copy (LSC) region (85,134 bp) (Fig. 1). All paired-end reads were mapped to the assembled cp genome with the mean coverage of 1,290.7. Coding regions (94,848, 59.31%) occupied over half of the cp genome, with the CDS (82,905 bp, 51.84%) regions forming the largest group, followed by rRNA genes (9,058 bp; 5.66%) and tRNA genes (2,885 bp; 1.80%). The remaining 40.71% is covered by intergenic regions, introns or pseudogenes (Table 1). The sequence of the chloroplast genome was deposited in GenBank (accession number: MF491390).

**Table 1:** Summary of the characteristics of *Fagopyrum dibotrys* chloroplast genome.

Feature	*F. dibotrys*
Total cpDNA size (bp)	159,919
LSC size (bp)	85,134
SSC size (bp)	13,309
IR size (bp)	30,738
Protein-coding regions (%)	51.83%
rRNA and tRNA (%)	7.47%
Introns size (% total)	10.73%
Intergenic sequences and pseudogenes (%)	27.54%
Number of genes	131
Number of different protein-coding genes	80
Number of different tRNA genes	28
Number of different rRNA genes	4
Number of different duplicated genes	18
Pseudogenes	1
GC content	37.9%

The *F. dibotrys* cp genome was predicted to contain 131 genes, including 80 protein-coding genes, 28 tRNA genes and 4 rRNA genes (Table 1). Among these genes, five protein-coding genes (*rpl2*, *ycf2*, *ndhB*, *rps7*, *ycf1*), seven tRNA genes and four rRNA genes (*rrn16*, *rrn2*3, *rrn4.5*, *rrn5*) were duplicated in IR regions. *rpl23*, which was repeated in the IR regions, was inferred to be a pseudogene. In the *F. dibotrys* cp genome, 18 genes contained introns, and 15 of them (9 peptide-coding genes and 6 tRNA genes) harbored one intron, whereas three genes (*rps12*, *clpP*, *ycf3*) harbored two introns. Of the 18 intron-containing genes, *rpl2*, *ndhB*, *rps12*, *trnI-GAU*, and *trnA-UGG* were located in the IR regions (Table 2). The *rps12* gene is a trans-spliced gene with its N-terminal exon located in the LSC region and the two remaining exons located in the IR regions. The *trnK-UUU* has the largest intron (2,484 bp) and includes the additional gene *matK*. The overall AT content of *F. dibotrys* cp genome is 62.1% and the corresponding values in LSC, SSC and IR regions are 63.8%, 67.2% and 58.7%, respectively. The frequency of codon usage was calculated for the cp genome based on the sequences of protein-coding genes and tRNA genes, which was summarized in Table 3. Similar to the phenomenon detected in other angiosperms cp genes, codon usage was biased toward a high representation of U and A at the third codon position^4,26^.

**Table 2:** Genes with introns in the *Fagopyrum dibotrys* chloroplast genome and the length of the exons and introns.

Gene	Location	exon I(bp)	intron I(bp)	exon II(bp)	intron II(bp)	exon III(bp)
*trn*K-UUU	LSC	37	2484	35		
*rps*16	LSC	40	847	227		
*trn*G*-*UCC	LSC	23	704	49		
*atp*F	LSC	144	753	411		
*rpo*C1	LSC	432	769	1611		
*ycf*3	LSC	124	865	116	754	153
*trn*L-UAA	LSC	53	503	50		
*trn*V-UAC	LSC	38	577	35		
*clp*P	LSC	71	1000	292	611	270
*pet*B	LSC	6	761	642		
*pet*D	LSC	8	727	475		
*rpl*16	LSC	9	1002	399		
*rpl*2	IR	393	662	435		
*ndh*B	IR	777	679	756		
*rps*12	IR	232	533	26		
*trn*I-GAU	IR	37	946	35		
*trn*A*-*UGC	IR	38	809	35		
*ndh*I	SSC	559	1018	539

**Table 3:** Codon–anticodon recognition pattern and codon usage for the *F. dibotrys* chloroplast genome.

Codon	Amino acid	Count	RSCU	tRNA	Codon	Amino acid	Count	RSCU	tRNA
UUU	F	2243	1.19	*trnF-GAA*	UAU	Y	1480	1.38	*trnY-GUA*
UUC	F	1526	0.81		UAC	Y	668	0.62	
UUA	L	1081	1.24	*trnL-UAA*	UAA	*	1279	1.27	
UUG	L	1112	1.28	*trnL-CAA*	UAG	*	814	0.81	
CUU	L	1098	1.26	*trnL-UAG*	CAU	H	928	1.4	*trnH-GUG*
CUC	L	687	0.79		CAC	H	397	0.6	
CUA	L	782	0.9		CAA	Q	1105	1.38	*trnQ-UUG*
CUG	L	461	0.53		CAG	Q	500	0.62	
AUU	I	1770	1.2	*trnI-GAU*	AAU	N	1785	1.36	*trnN-GUU*
AUC	I	1146	0.77		AAC	N	848	0.64	
AUA	I	1525	1.03	*trnI-CAU*	AAA	K	2278	1.35	*trnK-UUU*
AUG	M	907	1	*trnM-CAU*	AAG	K	1098	0.65	
GUU	V	832	1.35	*trnV-GAC*	GAU	D	983	1.37	*trnD-GUC*
GUC	V	490	0.79		GAC	D	455	0.63	
GUG	V	396	0.64		GAA	E	1299	1.37	*trnE-UUC*
GUA	V	750	1.22	*trnV-UAC*	GAG	E	595	0.63	
UCU	S	1107	1.4	*trnS-GGA*	UGU	C	690	1.19	*trnC-GCA*
UCC	S	888	1.12		UGC	C	472	0.81	
UCG	S	690	0.87		UGA	*	924	0.92	
UCA	S	832	1.05	*trnS-UGA*	UGG	W	737	1	*trnW-CCA*
CCU	P	699	1.09	*trnP-UGG*	CGU	R	405	0.72	*trnR-ACG*
CCC	P	588	0.92		CGC	R	275	0.49	*trnR-UCU*
CCA	P	802	1.25		CGA	R	611	1.09	
CCG	P	469	0.73		CGG	R	420	0.75	
ACU	T	729	1.19		AGA	R	1047	1.86	
ACC	T	609	1		AGG	R	615	1.09	
ACG	T	434	0.71	*trnT-GGU*	AGU	S	701	0.88	*trnS-GCU*
ACA	T	672	1.1	*trnT-UGU*	AGC	S	540	0.68	
GCU	A	526	1.27	*trnA-UGC*	GGU	G	584	0.99	*trnG-GCC*
GCC	A	391	0.94		GGC	G	386	0.65	
GCA	A	455	1.1		GGG	G	610	1.03	
GCG	A	290	0.7		GGA	G	790	1.33	*trnG-UCC*

### Repeat analysis

We identified 11 forward repeats, 26 palindromic repeats, and 16 tandem repeats in the *F. dibotrys* cp genome (Table S1). Most of the repeats (77.78%) were between 20 and 50 bp and 63.90% of repeats were located in intergenic spacer regions and introns. Within the CDS region, *ycf1* contained 4 tandem repeats, 5 palindromic repeats and 4 forward repeats, respectively (Table S1). Cp microsatellites (cpSSRs) are potentially useful markers for detection of polymorphisms in evolutionary studies of plants^27^. In the present study, a total of 48 SSR loci were detected for *F. dibotrys* cp genome, more than half of them (60.41%) were A and T mononucleotide repeats, followed by dinucleotide (22.91%), trinucleotide (8.33%) and tetranucleotide repeats (8.33%) (Table 4). Most SSRs were located in intergenic regions, but some of them were found in CDS regions such as *ycf1*, *matK, rpoB, rpoA, ycf2, rpoC2, ndhC, ndhD, cemA, rpl22, atpA* (Table 4).

**Table 4:** List of simple sequence repeats in *F. dibotrys*. The SSR-containing coding regions are indicated in parentheses.

Repeat unit	Length (bp)	Number	Start position
A	10	7	9,713; 14,788; 27,437; 36,949; 68,853; 87,979 (*ycf2*); 157,350
	11	7	15,751; 31,913; 44,655 (*ycf3*-intronII); 47,018; 50,586; 58,033; 79,049;
	13	1	55,533
	14	1	113,630 (*ycf1*)
T	10	9	3,362 (*matK*); 8,155; 8,335; 25,641 (*rpoB*); 49,786; 52,474; 79,293 (*rpoA*); 87,690; 157,061 (*ycf2*)
	11	2	17,926 (*rpoC2*); 51,300 (*ndhC*);
	12	1	84,892 (*rpl22*);
	14	1	131,411 (*ycf1*)
AT	5	2	46,915; 122,932 (*ndhD*)
	6	2	36,027; 78,305;
	7	2	115,630; 129,411
TA	5	2	45798; 63,096 (*petA*)
AAG	4	1	153,940 (*ycf2*)
ATA	4	1	123,501
CTT	4	1	91,098 (*ycf2*)
TTA	4	1	32,198
AATA	3	1	121,571 (*ndhD*)
AATG	3	1	62,815 (*cemA*)
AATT	3	1	14,007
GTCT	3	1	10,776 (*atpA*)

### Comparison of *F. dibotrys* to other Polygonaceae cp genomes

**Figure 2:**
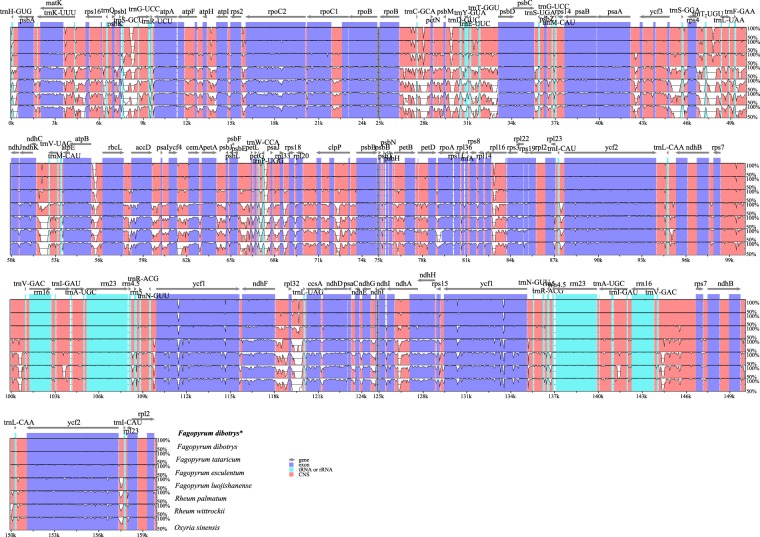
mVISTA percent identity plot comparing the eight Polygonaceae plastid genomes with *F. dibotrys* as a reference. The top line shows genes in order (transcriptional direction indicated by arrows). The y-axis represents the percent identity within 50–100%. The x-axis represents the coordinate in the chloroplast genome. Genome regions are color coded as protein-coding (exon), tRNA or rRNA, and conserved noncoding sequences (CNS). The asterisk indicated the cp genome of *F. dibotrys* obtained in the present study.

**Figure 3:**
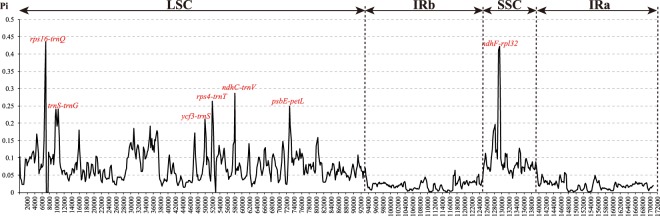
Nucleotide diversity (*Pi*) by sliding window analysis in the aligned whole cp genomes of seven Polygonaceae species. Window length: 600 bp, step size: 200 bp.

To understand the structural characteristics in cp genome of *F. dibotrys*, overall sequence alignment among seven Polygonaceae cp genomes were conducted using the annotation of *F. dibotrys* as a reference. The aligned chloroplast genome sequences were relatively conserved in seven Polygonaceae species, although some highly divergent regions were found. Similar to most angiosperm cp genomes, gene coding regions were more conserved than those of their noncoding counterparts (Fig. 2). Based on the alignment results, the most divergent non-coding regions among the eight cp genomes were *trnH*(GUG)*-psbA, rps16-trnQ*(UUG)*, psbI-trnS*(GCU)*, trnS*(GCU)-*trnG*(UCC), *petN-psbM, psbM-trnD*(GUC)*, trnE*(UUC)*-trnT*(GGU)*, atpB-rbcL, psaA-ycf3, ycf3-trnS*(GCA)*, rps4-trnT*(UGU)*, psbE-petL, ycf2-trnL*(CAA)*, ndhF-rpl32*. Slightly sequence variation was observed among eight cp genomes in the *atpF, rpoC2, rps19* and *ycf1* gene. Most of these hotspot regions located in the LSC regions and only few regions located in the SSC or IR region (Fig. 3). *Fagopyrum dibotrys* cp genome of the present study was divergent in some intergenic regions (including the above non-coding regions) compared with the previous study^25^. *F. dibotrys* and other five Polygonaceae species were used to validate the discriminatory powers of these highly variable regions. The results indicated that almost all primer pairs amplified PCR products with the expected fragment size (Fig. S1, Supplementary Dataset 1), and these loci were able to discriminate more than two species. Our results indicated that these variable regions could be used as new genetic markers for authentication and phylogeny in Polygonaceae species.

**Figure 4:**
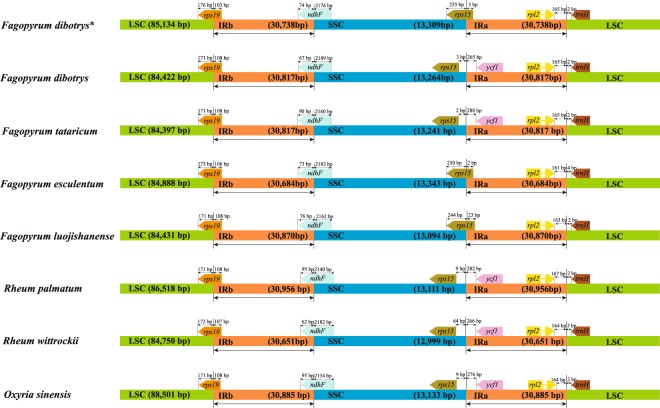
Comparison of chloroplast genome borders of LSC, SSC, and IRs among seven Polygonaceae species. The asterisk indicated the cp genome of *F. dibotrys* obtained in the present study.

Although genomic structure and size were relatively conserved in seven Polygonaceae cp genomes, the IR/SC boundary regions still varied slightly (Fig. 4). Five genes, including *rps19*, *ndhF*, *rps15*, *ycf1*, *rpl2* and *trnH*, were found in the junctions of LSC/IR and SSC/IR regions of eight cp genomes. Inconsistent with other cp genomes, only *ndhF* gene was detected across the IRb/SSC border in these seven species. *Rps15* was found to be 9 bp, 64 bp, 2 bp and 3 bp away from the SSC/IRa border in three Rumiceae species (*R. palmatum*, *O. sinensis* and *R. wittrockii*), *F. tataricum* and *F. dibotrys* (KY275181); but its 5′ end was extended 2 bp, 3 bp and 23 bp to the SSC/IRa border in *F. esculentum*, *F. dibotrys* and *F. luojishanense*, respectively (Fig. 4).

### Divergence of coding gene sequence

**Figure 5:**
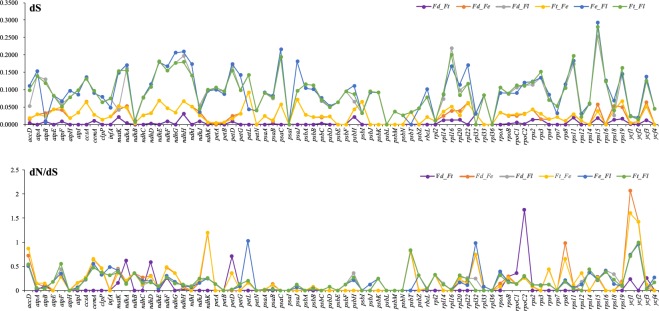
The dS and dN/dS values of 78 protein-coding genes from four *Fagopyrum* cp genomes (*Fd*: *F. dibotrys*; *Fe*: *F. esculentum* subsp. *ancestrale*; Ft: *F. tataricum*; Fl: *F. luojishanense*).

To detect the selective pressure on the 78 cp genes of four *Fagopyrum* species. We calculated the rates of synonymous (dS) and nonsynonymous (dN) substitutions and the dN/dS ratio (Fig. 5). The average dS values between paired *Fagopyrum* species (*F. dibotrys* vs *F. tataricum*/*F. dibotrys* vs *F. esculentum* subsp. *ancestrale*/*F. dibotrys* vs *F. luojishanense*/*F. tataricum* vs *F. esculentum* subsp. *ancestrale/F. esculentum* subsp. *ancestrale* vs *F. luojishanense*/*F. tataricum* vs *F. luojishanense*) were 0.0038/0.0236/0.0840/0.0241/0.0873/0.0873, 0.0085/0.0511/0.1571/0.0489/0.1724/0.1547 and 0.0002/0.0089/0.0215/0.0091/0.0190/0.0217 in the LSC, SSC, and IR regions respectively, with a total average value of 0.0042/0.0266/0.0903/0.0266/0.0949/0.0926 across all regions (Table S2). The dN values ranged from 0 to 0.0640, with a total average value of 0.0010/0.0053/0.0148/0.0055/0.0159/0.0148 across all whole cp genomes. Most dN/dS ratios were less than 1, possibly indicating that cpDNA genes were under purifying selection. Only five genes (*ndhK, petL, rpoC2, ycf1, ycf2*) had dN/dS values >1, indicating that these genes had undergone positive selection (Table S2).

### Phylogenetic analysis

**Figure 6:**
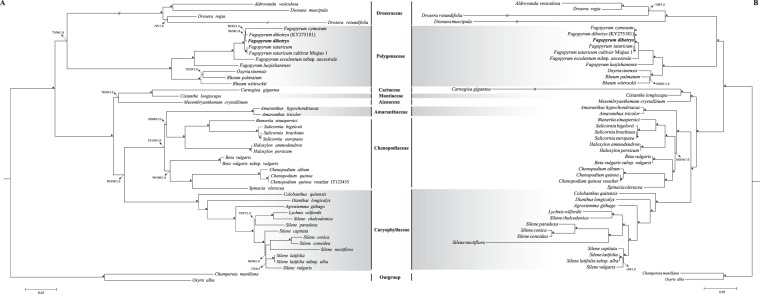
Phylogenetic tree reconstruction of 42 taxa using maximum likelihood, maximum parsimony and Bayesian inference based on datasets of the 50 shared genes and entire genome sequence. (**A**) The dataset of 50 shared genes. (**B**) The entire genome sequence dataset. ML topology was shown with ML bootstrap value/MP bootstrap value /Bayesian posterior probability given at each node. The Pentastar in the phylogenetic tree indicated that the support rate of branch is 100/100/1.0.

In the present study, complete cp genomes and 50 shared cp genes shared among order Caryophyllales were utilized to depict the phylogenetic relationships. Phylogenetic analyses were performed using maximum parsimony (MP), maximum likelihood (ML) and Bayesian inference (BI) methods. Two Santalales species, *Osyris alba* and *Champereia manillana* were set as outgroup. The dataset comprised of 382,668/39,085 (cp genomes/50 cp genes) nucleotide positions with 73,706/8,726 informative sites. The results of ML analyses based on two different datasets (i.e. complete cp genomes and 50 shared genes) were showed in Fig. 6, which shared identical topology of phylogenetic tree inferred from the MP and BI analysis. The Pentastar in the phylogenetic tree indicated that the support rate of branch was 100/100/1.0. The results showed same phylogenetic signals for the complete cp genomes and 50 shared genes of species in order Caryophyllales, and only a few species showed inconsistent interspecific relationships based on these two datasets (Fig. 6A,B). Our phylogenetic trees supported the monophyly of order Caryophyllales and three families including Droseraceae, Polygonaceae and Caryophyllaceae also formed a monophyletic clade with high bootstrap values (MP and ML analyses) and posterior probability value (BI analysis). Interestingly, two Amaranthaceae species clustered in the same clade were embedded in the family Chenopodiaceae, which corroborated the close relationship between these two families^28^. We found all the *Fagopyrum* species formed a monophyletic clade with high resolution, and *F. dibotrys* was placed along with *F. tataricum*.

## Discussion

In this study, the complete cp genome of *Fagopyrum dibotrys* was assembled by using Illumina sequencing reads derived from the whole genome. This strategy without prior isolation of the cpDNA, provided a new way to obtain the cp genome and had been successful in many studies^4,29–31^. The cp genome will provide a series of resources for evolutionary and genetic studies about this endangered medicinal plant.

The cp genome of *F. dibotrys* possess the typical angiosperm quadripartite structure with two short inverted repeat regions separated by two single copy regions (Fig. 1) and the gene content with a size in range with other Polygonaceae species^3,32,33^. Notably, we found the newly sequenced cp genome of *F. dibotrys* was almost identical with the previously published one^25^, and the sequence divergences of these two genomes were mainly distributed in non-coding regions (*trnS-trnG*, *rpoB-trnC*, *psbM-trnD*, *ndhC-trnV, atpB-rbcL, trnP-psaJ*). Although cp genome is remarkably conserved relative to gene content, some variable regions that include insertions/deletions could be detected^34^. Therefore, some variable regions were found in the two cp genomes of *F. dibotrys*. According to the alignment result, no significant structural rearrangements, such as inversions or gene relocations were detected in these eight cp genomes. The eight plastomes of Polygonaceae were relatively well conserved, and most variations were detected in intergenic regions (Fig. 2). DNA barcodes are defined as the DNA sequences with a sufficiently high mutation rate to identify a species within a given taxonomic group and are confirmed as reliable tools for the identification of medicinal plants^35,36^. Here, highly variable in regions such as *atpF, rpoC2, rps19* and *ycf1, trnH*(GUG)*-psbA, rps16-trnQ*(UUG)*, psbI-trnS*(GCU)*, trnS*(GCU)-*trnG*(UCC) *petN-psbM, psbM-trnD*(GUC)*, trnE*(UUC)*-trnT*(GGU)*, atpB-rbcL, pasA-ycf3, ycf3-trnS*(GCA)*, rps4-trnT*(UGU)*, psbE-petL, ycf2-trnL*(CAA)*, ndhF-rpl32* were detected. As our results showed, most of them located in LSC region and these regions can discriminate some Polygonaceae species successfully (Fig. S1). Therefore, the above highly variable regions could be used as specific DNA barcodes for authentication of the source plant in family Polygonaceae, and these regions also provide sufficient genetic markers for resolving the phylogeny of family Polygonaceae.

The contraction and expansion at the borders of the IR regions are the main reasons for the size variation of cp genomes^37^. Despite the similar lengths of the IR regions of *F. dibotrys* and the other Polygonaceae species, some expansion and contraction were observed, with the IR regions ranging from 30,651 bp in *R. wittrockii* to 30,956 bp in *R. palmatum*. In this study, only *ndhF* gene was detected across the IRb/SSC border in seven Polygonaceae species, which was caused by a duplication of the normally single-copy gene *ycf1*. In general, *ycf1*, which was located in IRb, is considered a pseudogene in several angiosperm cp genomes. However, no stop codons were detected in the coding sequence of *ycf1*, thus the long length of *ycf1* affected the differences of gene distribution at the SC/IR borders. We deduced that the expansion of the IR caused a duplication of *ycf1*. Gene duplications caused an expansion of the IR in *Eucommia ulmoides* as well^38^.

Repeat elements are correlated with plastome rearrangement and recombination^39,40^. In this study, a low number of repeats was detected in the *F. dibotrys* cp genome, and most repeats were located in intergenic regions or in *ycf1*. Repeats in the *ycf1* gene are commonly observed^41^. Most of the repeated regions identified in the present study showed similar characteristics to the congeneric species^3^. Cp microsatellites (cpSSRs) usually showed high variation within the same species and which are potentially useful markers for population genetics^27^. In this study, some SSRs were identified that could be used to infer the population genetic structure and help to develop more conservation strategies for *F. dibotrys*. These SSR markers also be useful for genetic diversity studies of other Polygonaceae species.

Sequence divergence of protein coding genes was evaluated by calculating the synonymous (dS) substitution rates; all of the genes showed a low sequence divergence (dS < 0.1). Our analyses indicated that most cp genes were under purifying selection (dN/dS < 1); similar results were reported for other cp genomes^30,42,43^. Only five genes (*ndhK*, *petL*, *rpoC2*, *ycf1*, *ycf2*) had dN/dS ratio >1 as expected of genes under positive selection. Eleven genes in plant cp genome (*ndhA*-*ndhK*) encode NAD(P)H dehydrogenase (NDH) complex which plays important role in photosystem I cyclic electron transport and chlororespiration^44,45^. Because the NDH monomer is sensitive to high light intensity, we deduced that the genes encoded NAD(P)H dehydrogenase might have changed drastically to develop new functions for stress resistance^45,46^. Previous research reported that genes belong to subunits of cytochrome were under positive selection in some species^47,48^, we therefore inferred that *petL* for cytochrome b6/f complex subunit proteins may have a high evolution rate in the cp genome of *F. luojishanense*. The gene *rpoC2* was associated with PPR7 protein, we thus speculated it may have coevolved with nuclear genes^49^. The *ycf1* and *ycf2* are two of the largest genes encoding for a putative membrane protein^50,51^ and in two *Fagopyrum* cp genomes these two genes may have rapidly evolved^3^.

Cp genomes with sufficient informative sites have been proven to be effective in resolving difficult phylogenetic relationships^7,8^. Until now, the phylogeny of Caryophyllales was analyzed using only a few genetic markers, and the phylogenetic positon of *F. dibotrys* is still needed to be clarified. Here, the phylogeny of the Caryophyllales was rebuilt using MP, ML, Bayesian methods based on complete cp genomes and 50 shared PCGs. Phylogenetic trees inferred from different methods showed an identical topology with high resolution values at most clades. And trees rebuilt based on complete cp genomes and 50 shared genes also showed identical topology except some Droseraceae species, which was mainly caused by the unusual structure, plastome-wide rearrangements and gene losses in Droseraceae cp genomes. We thus presumed that shared genes may provide more reliable phylogenetic signals for the species with unusual structure of cp genome. In our study, species of the Polygonaceae formed a monophyletic clade and showed a paraphyletic relationship with species in the Droseraceae, which was consistent with the previous phylogenetic study based on *rbcL* and *matK*^52^. Two species from Cactaceae and Aizoaceae species showed a paraphyletic relationship, which was in accordance with the phylogeny inferred from cpDNA^53^. Our phylogenetic analyses provided robust support for the monophyly of species in the Amaranthaceae, Chenopodiaceae and Caryophyllaceae; previous studies of the phylogeny of the Caryophyllales resulted in similar findings, but with relatively low support values^53^. Unexpectedly, two species of Amaranthaceae were clustered with the Chenopodiaceae species, indicating a close relationship between these two taxa. Previous phylogenetic and morphological research showed that Amaranthaceae and Chenopodiaceae were closely related families and had long been considered a single evolutionary lineage^28^. Therefore, our study further confirmed the close relationships of these two families. We found that all *Fagopyrum* species formed one monophyletic clade along with three Rumiceae species, and *F. dibotrys* was related to *F. tataricum*, as in the phylogeny reported by Zhou *et al*.^54^ using ITS and *matK*. Our phylogeny based on cp genomes further confirmed that *F. dibotrys* is not the ancestor of cultivated buckwheat and *F. dibotrys* is closer to Tartary buckwheat than to common buckwheat^20–22^. Although our results clarified the phylogenetic relationships of some Caryophyllales species based on the available cp genomes, more complete cp genome sequences are need to resolve the comprehensive phylogenies of this order, especially since limited taxon sampling may produce discrepancies in tree topologies^4,55^.

## Conclusions

Our study reported the complete chloroplast genome of *Fagopyrum dibotrys*, which provided valuable plastid genomic resources for this endangered medicinal plant. The cp genome organization and gene content are similar to that of congeneric species. We also identified SSRs that could be used for population genetics studies within *Fagopyrum*. The comparative analysis of the genome structure of seven Polygonaceae plants showed several variation hotspots, which could be used to develop more specific DNA barcodes for the authentication of Polygonaceae speies. And these highly variable regions also presented a solid resource for phylogenetic studies in the family Polygonaceae. Coding gene sequence divergence analyses indicated that only a few genes were subject to positive selection. We depicted the phylogenetic relationships of some species belong to order Caryophyllales and confirmed the phylogenetic relationship between *F. dibotrys* and common buckwheat.

## Materials and Methods

### Plant material

Young leaves of *F. dibotrys* were collected from Pingli, Shaanxi, China (32°23′33″N, 109°21′61″E). Voucher specimen of *F. dibotrys* was deposited at Xi’an Botanical Garden Herbarium (XBGH).

### Chloroplast genome sequencing, assembly and annotation

Total genomic DNA was extracted from the fresh leaves of *F. dibotrys* using a CTAB-based protocol^56^. The DNA library was prepared according to the method of Zhou *et al*.^30^ and then a paired-end library was sequenced using Illumina hiseq^TM^ 2500 platform with the average read length of 125 bp. The raw reads were trimmed using NGS QC Toolkit_v2.3.3 with default cut-off values^57^. After trimming of low quality reads and adapters, the clean reads were mapped to the cp genome of *F*. *esculentum* subsp. *ancestrale* (EU254477) using Bowtie 2–2.2.6 with default values^58^. The matched paired-end reads were assembled using SPAdes-3.6.0^59^. After *de novo* assembly, some ambiguous regions were picked out to extend length with MITObim v1.8^60^. Eventually, the complete chloroplast genome was annotated using DOGMA^61^ and the primary annotated results were manually verified according to the reference cp genome in Geneious R9 v 9.0.2 (Biomatters Ltd., Auckland, New Zealand). The circular plastid genome map was completed using the online program OrganellarGenome DRAW^62^

### Genome analysis, codon usage, repeat structure and sequence divergence

Whole chloroplast gene distribution of all seven Polygonaceae species was performed and visualized using mVISTA software with the annotation of *F. dibotrys* as a reference^63^. The nucleotide diversity (*Pi*) and sequence polymorphisms of seven Polygonaceae species were analyzed using DNAsp 6.0^64^. In order to validate the divergence hotspot regions and develop specific DNA barcodes for discriminating species in Polygonaceae. The primer pairs were designed based on the sequence of *F. dibotrys* cp genome (Table S3) and validated using the genomic DNA of *F. dibotrys* and other 5 Polygonaceae species including *Rumex crispus, Rheum hotaoense, Reynoutria japonica, Rheum palmatum*, and *Fallopia multiflora*. PCR amplification to validate these hotspot regions were performed in a reaction volume of 25 μL with 12.5 μL 2 × Taq PCR Master Mix, 0.4 μM of each primer, 2 μL template DNA and 10.1 μL ddH_2_O. All amplifications were carried out in SimpliAmp™ Thermal Cycler (Applied Biosystems, Carlsbad, CA,USA) as follow: denaturation at 94 °C for 5 min, followed by 35 cycles of 94 °C for 50 s, at specific annealing temperature (Tm) for 45 s, 72 °C for 90 s and 72 °C for 7 min as final extension. PCR products were visualized on 2% after staining with agarose gels ethidium bromide and then the DNA fragments were sequenced by Sangon Biotech (Shanghai, China).

The codon usage frequency was calculated by using MEGA6^65^. Dispersed and palindromic repeats of *F. dibotrys* cp genome were identified using REPuter with a minimum repeat size of 30 bp and a sequence identity >90%^66^. Tandem repeat sequences were searched using the Tandem Repeats Finder program with the following parameters: 2 for alignment parameters match, 7 for mismatch and indel, respectively^67^. Simple sequence repeats (SSRs) were analyzed using MISA (http://pgrc.ipk-gatersleben.de/misa/) with the parameters of ten for mono, five for di-, four for tri-, and three for tetra-, penta, and hexa-nucleotide motifs. In order to detect whether plastid genes were under selection pressure, the nonsynonymous (dN), synonymous (dS), and dN/dS values of each protein coding gene in the three *Fagopyrum* cp genomes were analyzed using PAML packages 4.0 with YN algorithm^68^.

### Phylogenetic analysis

In this study, 45 cp genomes available in GenBank were recovered to infer the phylogenetic relationships among 42 species belonging to the order Caryophyllales. *Osyris alba* and *Champereia manillana* were set as out-group (Table S4). First, multiple alignments were performed using complete cp genomes based on the conserved structure and gene order of the chloroplast genomes. All the nucleotide sequences were aligned using MAFFT v7.308^69^ with default parameters. Three methods were employed to construct phylogenetic trees, including maximum parsimony (MP), maximum likelihood (ML) and Bayesian inference (BI). Maximum parsimony (MP) analyses were performed using PAUP 4.0b10^70^ and addition-sequence was set as 1,000 replications for Heuristic search. The Maximum likelihood (ML) analyses were conducted using IQ-TREE^71^ with the best best-fit model selected by ModelFinder in the IQ-TREE package^72^ (Table S5) and the bootstrap replicates were 1,000. Bayesian inference (BI) was conducted using MrBayes v3.2.6^73^ with the nucleotide substitution model inferred from Modeltest 3.7^74^ (Table S5). The Markov chain Monte Carlo (MCMC) algorithm was run for 2 million generations and sampled every 100 generations. The first 25% of trees generated were discarded as burn-in and the remaining trees were used to build a majority-rule consensus tree with posterior probability (PP) values for each node. Due to gene loss, inversion and unusual structure were detected in the cp genomes of some species (e.g. *Carnegiea gigantea*, *Dionaea muscipula* and *Drosera rotundifolia*). The above three phylogenetic-inference methods were used to infer the phylogenetic tree from 50 shared cp genes using the same settings (Table S6).

### Data availability

The complete chloroplast sequence generated and analyzed during the current study are available in GenBank, https://www.ncbi.nlm.nih.gov/genbank/ (accession numbers are described in the text).

## Electronic supplementary material

Supplementary Information

Dataset 1
