# Supplementary material for: Complete chloroplast genome sequence of Fagopyrum dibotrys: genome features, comparative analysis and phylogenetic relationships
Source: Sci Rep. 2018 Aug 17;8:12379. doi: 10.1038/s41598-018-30398-6 (PMC6098159; doi:10.1038/s41598-018-30398-6)
Supplement: Supplementary file 1 — Supplementary Information [file 41598_2018_30398_MOESM1_ESM.pdf]

Complete chloroplast genome sequence of *Fagopyrum dibotrys*: genome features, comparative analysis and phylogenetic relationships

Xumei Wang[1,*,+], Tao Zhou[1,+], Guoqing Bai[2], Yuemei Zhao[3]

1 School of Pharmacy, Xi'an Jiaotong University, Xi'an 710061, China

2 Shaanxi Engineering Research Centre for Conservation and Utilization of Botanical Resources, Xi'an Botanical Garden of Shaanxi Province, Xi'an 710061, China

3 College of Biopharmaceutical and Food Engineering, Shangluo University, Shangluo 726000, China

*Correspondence.

E-mail: wangxumei@mail.xjtu.edu.cn

[+] These authors contributed equally to this article.

Supplementary Tables

Table S1 Repeated sequences in the *Fagopyrum dibotrys* chloroplast genome.

Table S2 dN/dS ratio between pairwise of species protein coding sequences.

Table S3 The primers designed for divergence hotspot regions amplification

Table S4 List of plastome sequences included in the phylogenetic analyses

Table S5 Model in ML and BI analyses based on different datasets

Table S6 List of the 50 genes used in the phylogenetic analysis

Table S1 Repeated sequences in the *Fagopyrum dibotrys* chloroplast genome.

Start of the first repeat	Start of the second repeat	Type	Repeat size	Copy number	Location	Region
27,880	27,948	F	69	(×2)	IGS (*trnG-GCA-petN*)	LSC
119,594	119,659	F	66	(×2)	IGS (*rpl32-trnL-UAG*)	SSC
47,705	47,760	F	65	(×2)	IGS (*trnT-UGU-trnL-UAA*)	LSC
111,417	133,517	P	56	(×2)	*ycf1*	IRB,IRA
111,432	111,453	F	59	(×2)	*ycf1*	IRB
82,846	82,895	F	45	(×2)	*rpl16* intron I	LSC
111,470	133,520	P	42	(×2)	*ycf1*	IRB,IRA
111,446	111,467	F	45	(×2)	*ycf1*	IRB
111,500	133,480	P	37	(×2)	*ycf1*	IRB,IRA
111,449	133,517	P	45	(×2)	*ycf1*	IRB,IRA
111,428	111470	F	42	(×2)	*ycf1*	IRB

57,741	57,741	P	43	(×2)	IGS (*rbcL-accD*)	LSC
43,964	99,250	F	39	(×2)	*ycf3* intron I; *rps12* intron I	LSC; IRB
43,964	126,106	F	39	(×2)	*ycf3* intron I; *ndhA* intron I	LSC; SSC
43,964	145,761	P	39	(×2)	*ycf3* intron I; IGS (*trnV-GAC-rps12*)	LSC; IRA
67,053	67,053	P	39	(×2)	IGS (*trnW-CCA-trnP-UGG*)	LSC
7,555	45,696	P	30	(×2)	IGS *(psbI-trnS-GCU*); *trnS-GCA*	LSC
15,821	15,848	F	32	(×2)	IGS (*rps2-rpoC2*)	LSC
114,642	114,666	F	33	(×2)	*ycf1*	IRB
114,642	130354	P	33	(×2)	*ycf1*	IRB,IRA
3,693	3,740	T	24	(×2)	IGS (*trnS-GCU- trnG-UCC*)	LSC
13,660	13,703	T	22	(×2)	IGS (*atpH-atpI*)	LSC
15,822	15,877	T	27	(×2)	IGS (*rps2-rpoC2*)	LSC
27,127	27,174	T	22	(×2)	IGS (*rpoB-trnC-GCA*)	LSC
27,881	28,017	T	68	(×2)	IGS (*trnC-GCA-petN*)	LSC

27,904	28,012	T	34	(×3)	IGS (*trnC-GCA-petN*)	LSC
47,006	47,078	T	36	(×2)	IGS (*rps4-trnT-UGU*)	LSC
47,706	47,825	T	55	(×2)	IGS (*trnT-UGU- trnL-UAA*)	LSC
82,847	82,940	T	49	(×2)	*rpl16* intron I	LSC
108,211	108,276	T	32	(×2)	IGS (*rrn4.5-rrn5*)	IRB
111,429	111,512	T	21	(×4)	*ycf1*	IRB
114,643	114,695	T	24	(×2)	*ycf1*	IRB
119,595	119,725	T	65	(×2)	IGS (*rpl32-trnL-UAG*)	SSC
130,359	130,411	T	24	(×2)	*ycf1*	IRA
133,518	133,558	T	21	(×2)	*ycf1*	IRA
145,549	145,596	T	21	(×2)	IGS (*trnV-GAC-rps12*)	IRA

Note: F, forward; P, palindromic; T, Tandem; IGS, intergenic spacer region

Table S2 dN/dS ratio between pairwise of species protein coding sequences.

	FD_FT			FD_FE			FD_FL		
	dN	dS	dN/dS	dN	dS	dN/dS	dN	dS	dN/dS
*accD*_CDS	0.0018±0.0013	0.0036±0.0036	0.5063	0.0156±0.0038	0.0156±0.0038	0.726	0.0531±0.0071	0.0531±0.0071	0.5131
*atpA*_CDS	0	0	/	0.0042±0.0019	0.0283±0.0095	0.1491	0.0076±0.0025	0.1388±0.0231	0.0548
*atpB*_CDS	0.0009±0.0009	0.0080±0.0046	0.1115	0.0027±0.0016	0.0327±0.0095	0.0822	0.0036±0.0018	0.1289±0.0201	0.0278
*atpE*_CDS	0	0	/	0	0.0426±0.0215	0	0.0150±0.0071	0.0821±0.0305	0.1832
*atpF*_CDS	0.0023±0.0023	0.0081±0.0082	0.2872	0.0118±0.0053	0.0409±0.0184	0.289	0.0262±0.0079	0.0593±0.0228	0.441
*atpH*_CDS	0	0		0	0.0178±0.0179	0	0	0.0776±0.0394	0
*atpI*_CDS	0	0	/	0.0054±0.0031	0.0333±0.0137	0.1627	0.0036±0.0026	0.0982±0.0245	0.0368
*ccsA*_CDS	0.0053±0.0026	0	/	0.0146±0.0044	0.0645±0.0189	0.2261	0.0317±0.0066	0.1319±0.0287	0.2402
*cemA*_CDS	0	0.0110±0.0078	0	0.0181±0.0060	0.0279±0.0125	0.648	0.0457±0.0098	0.0963±0.0241	0.4741
*clpP*_CDS	0	0	/	0.0063±0.0036	0.0136±0.0096	0.4643	0.0233±0.0071	0.0636±0.0215	0.3668
*infA*_CDS	0	0	/	0	0.0227±0.0228	0	0.0233±0.0135	0.0742±0.0438	0.3136
*matK*_CDS	0.0033±0.0016	0.0206±0.0085	0.159	0.0190±0.0040	0.0420±0.0123	0.4523	0.0604±0.0072	0.0419±0.0239	0.4255
*ndhA*_CDS	0.0024±0.0017	0.0039±0.0039	0.6204	0.0097±0.0034	0.0522±0.0147	0.1858	0.0235±0.0054	0.1604±0.0274	0.1465
*ndhB*_CDS	0	0	/	0.0009±0.0009	0.0025±0.0025	0.361	0.0027±0.0015	0.0074±0.0043	0.3589
*ndhC*_CDS	0.0036±0.0036	0	/	0.0071±0.0050	0.0264±0.0188	0.2696	0.0089±0.0056	0.0762±0.0331	0.1162
*ndhD*_CDS	0.0017±0.0012	0.0029±0.0029	0.5922	0.0096±0.0029	0.0322±0.0098	0.2996	0.0198±0.0042	0.1075±0.0189	0.1846
*ndhE*_CDS	0	0	/	0.0042±0.0042	0.0684±0.0348	0.0607	0.0165±0.0083	0.1820±0.0653	0.0906
*ndhF*_CDS	0.0023±0.0011	0.0087±0.0044	0.2595	0.0224±0.0036	0.0471±0.0105	0.4743	0.0417±0.0050	0.1549±0.0206	0.2691
*ndhG*_CDS	0	0	/	0.0124±0.0056	0.0342±0.0173	0.3615	0.0252±0.0080	0.1760±0.0434	0.1433
*ndhH*_CDS	0.0011±0.0011	0.0300±0.0107	0.0367	0.0055±0.0025	0.0661±0.0163	0.0835	0.0240±0.0052	0.1975±0.0310	0.1213
*ndhI*_CDS	0	0	/	0	0.0513±0.0232	0	0.0122±0.0055	0.1409±0.0434	0.0865
*ndhJ*_CDS	0	0.0088±0.0088	0	0.0088±0.0088	0.0358±0.0180	0.1557	0.0112±0.0056	0.0544±0.0225	0.2064

*ndhK*_CDS	0	0	/	0.0071±0.0035	0.0059±0.0059	1.1964	0.0252±0.0068	0.0993±0.0253	0.2536
*petA*_CDS	0	0.0045±0.0045	0	0	0	/	0.0137±0.0044	0.1004±0.0225	0.1367
*petB*_CDS	0	0	/	0	0.0062±0.0062	0	0	0.0951±0.0265	0
*petD*_CDS	0.0057±0.0040	0.0080±0.0080	0.7071	0	0.0240±0.0140	0	0.0028±0.0028	0.1667±0.0420	0.0169
*petG*_CDS	0	0	/	0	0.0309±0.0310	0	0.0128±0.0129	0.0987±0.0580	0.1301
*petL*_CDS	0	0	/	0.0145±0.0146	0.0919±0.0665	0.1579	0.0294±0.0209	0.1418±0.0861	0.2076
*petN*_CDS	0	0	/	0	0	/	0	0.0397±0.0399	0
*psaA*_CDS	0	0	/	0	0.0241±0.0067	0	0.0012±0.0008	0.0903±0.0137	0.013
*psaB*_CDS	0	0.0019±0.0019	0	0.0012±0.0009	0.0093±0.0042	0.129	0.0012±0.0009	0.0753±0.0126	0.016
*psaC*_CDS	0	0	/	0	0.0577±0.0337	0	0	0.1945±0.0695	0
*psaI*_CDS	0.0121±0.0121	0	/	0	0	/	0.0121±0.0122	0	/
*psaJ*_CDS	0	0	/	0	0.0720±0.0421	0	0.0115±0.0115	0.0961±0.0492	0.1198
*psbA*_CDS	0	0	/	0	0.0278±0.0114	0	0	0.1148±0.0249	0
*psbB*_CDS	0	0	/	0.0017±0.0012	0.0207±0.0079	0.0819	0.0042±0.0019	0.1125±0.0199	0.0377
*psbC*_CDS	0	0.0028±0.0028	0	0	0.0197±0.0075	0	0	0.0671±0.0142	0
*psbD*_CDS	0	0	/	0	0.0223±0.0092	0	0.0038±0.0022	0.0493±0.0139	0.0776
*psbE*_CDS	0	0	/	0	0	/	0	0.0634±0.0324	0
*psbF*_CDS	0	0	/	0	0	/	0.0121±0.0121	0.0953±0.0561	0.1268
*psbH*_CDS	0	0.0213±0.0214	0	0	0.0431±0.0308	0	0.0238±0.0120	0.0653±0.0384	0.3649
*psbI*_CDS	0	0	/	0	0.0648±0.0466	0	0	0.0324±0.0330	0
*psbJ*_CDS	0	0	/	0.0118±0.0118	0	/	0	0.0947±0.0562	0
*psbK*_CDS	0	0	/	0	0	/	0.0224±0.0130	0.0913±0.0471	0.2459
*psbL*_CDS	0	0	/	0	0	/	0	0	/
*psbM*_CDS	0	0	/	0	0	/	0	0.0367 ± 0.0369	0
*psbN*_CDS	0	0	/	0	0	/	0	0.0262±0.0263	0
*psbT*_CDS	0	0	/	0.0291±0.0207	0.0354±0.0356	0.8235	0.0293±0.0209	0.0351±0.0353	0.8338

*psbZ*_CDS	0	0	/	0	0	/	0.0143±0.0102	0.0461±0.0328	0.3107
*rbcL*_CDS	0.0018±0.0013	0	/	0.0009±0.0009	0.0209±0.0079	0.0442	0.0028±0.0016	0.0782±0.0160	0.0353
*rpl2*_CDS	0	0	/	0.0032±0.0023	0.0099±0.0070	0.3264	0.0032±0.0023	0.0099±0.0070	0.3243
*rpl14*_CDS	0	0.0119±0.0119	0	0	0.0237±0.0169	0	0.0108±0.0062	0.0734±0.0304	0.147
*rpl16*_CDS	0	0.0126±0.0127	0	0	0.0386±0.0226	0	0.0031±0.0031	0.2188±0.0608	0.0141
*rpl20*_CDS	0	0.0128±0.0129	0	0.0065±0.0046	0.0392±0.0228	0.1644	0.0261±0.0093	0.0974±0.0381	0.2679
*rpl22*_CDS	0.0029±0.0029	0	/	0.0116±0.0058	0.0612±0.0253	0.1897	0.0307±0.0096	0.1165±0.0376	0.2638
*rpl32*_CDS	0	0.0300±0.0303	0	0.0227±0.0132	0.0305±0.0307	0.7435	0.0075±0.0075	0.0305±0.0307	0.2458
*rpl33*_CDS	0	0	/	0.0068±0.0068	0	/	0	0.0834±0.0429	0
*rpl36*_CDS	0	0	/	0	0	/	0	0	/
*rpoA*_CDS	0.0050±0.0025	0	/	0.0050±0.0025	0.0342±0.0131	0.1474	0.0392±0.0072	0.1058±0.0240	0.3705
*rpoB*_CDS	0.0004±0.0004	0.0014±0.0014	0.2922	0.0073±0.0017	0.0250±0.0059	0.2919	0.0153±0.0025	0.0873±0.0116	0.1747
*rpoC1*_CDS	0.0018±0.0011	0.0051±0.0036	0.3603	0.0034±0.0014	0.0245±0.0080	0.138	0.0145±0.0030	0.1060±0.0177	0.1371
*rpoC2*_CDS	0.0019±0.0008	0.0011±0.0011	1.6719	0.0083±0.0016	0.0305±0.0060	0.2727	0.0343±0.0034	0.1124±0.0123	0.3055
*rps2*_CDS	0	0.0140±0.0099	0	0.0036±0.0025	0.0430±0.0177	0.0826	0.0143±0.0051	0.1146±0.0311	0.125
*rps3*_CDS	0	0.0149±0.0106	0	0	0.0151±0.0107	0	0.0155±0.0055	0.1347±0.0349	0.1148
*rps4*_CDS	0	0	/	0.0066±0.0038	0.0149±0.0106	0.4418	0.0088±0.0044	0.0699±0.0236	0.1256
*rps7*_CDS	0	0	/	0	0.0205±0.0145	0	0	0.0529±0.0242	0
*rps8*_CDS	0.0033±0.0033	0	/	0.0100±0.0058	0.0102±0.0102	0.9802	0.0216±0.0085	0.1053±0.0350	0.2056
*rps11*_CDS	0	0.0193±0.0137	0	0.0032±0.0032	0.0291±0.0170	0.1114	0.0065±0.0046	0.1737±0.0455	0.0373
*rps12*_CDS	0.0037±0.0037	0	/	0	0.0102±0.0103	0	0.0037±0.0037	0.0207±0.0147	0.179
*rps14*_CDS	0.0131±0.0076	0	/	0	0	/	0.0221±0.0099	0.0593±0.0300	0.3725
*rps15*_CDS	0.0052±0.0052	0.0184±0.0184	0.2821	0.0155±0.0090	0.0581±0.0339	0.2673	0.0584±0.0178	0.2519±0.0828	0.2316
*rps16*_CDS	0.0046±0.0046	0	/	0.0185±0.0093	0	/	0.0521±0.0159	0.1233±0.0584	0.4227
*rps18*_CDS	0	0.0130±0.0131	0	0	0.0536±0.0271	0	0.0089±0.0063	0.0264±0.0188	0.3368
*rps19*_CDS	0	0.0159±0.0159	0	0.0094±0.0067	0.0500±0.0293	0.1883	0.0141±0.0082	0.1436±0.0523	0.0984

	FT_FE			FE_FL			FT_FL		
*ycf1*_CDS	0.0002±0.0002	0.0009±0.0009	0.2349	0.0078±0.0013	0.0038±0.0019	2.0678	0.0168±0.0019	0.0224±0.0046	0.7485
*ycf2*_CDS	0.0008±0.0004	0	/	0.0112±0.0015	0.0079±0.0024	1.4247	0.0150±0.0018	0.0150±0.0033	0.9992
*ycf3*_CDS	0.0032±0.0032	0.0125±0.0125	0.2596	0.0032±0.0032	0.0642±0.0290	0.0506	0.0065±0.0046	0.1378±0.0452	0.047
*ycf4*_CDS	0	0	/	0.0049±0.0034	0	/	0.0073±0.0042	0.0443±0.0182	0.1652

	FT_FE			FE_FL			FT_FL		
	dN	dS	dN/dS	dN	dS	dN/dS	dN	dS	dN/dS
*accD*_CDS	0.0156±0.0038	0.0178±0.0080	0.876	0.0562±0.0074	0.1104±0.0217	0.5096	0.0531±0.0071	0.0989±0.0204	0.5369
*atpA*_CDS	0.0042±0.0019	0.0283±0.0095	0.1491	0.0067±0.0024	0.1533±0.0249	0.0439	0.0076±0.0025	0.1388±0.0231	0.0548
*atpB*_CDS	0.0036±0.0018	0.0243±0.0081	0.1481	0.0063±0.0024	0.0063±0.0024	0.0505	0.0045±0.0020	0.1186±0.0191	0.0378
*atpE*_CDS	0	0.0426±0.0215	0	0.0150 ± 0.0071	0.0826± 0.0306	0.1817	0.0150 ± 0.0071	0.0821± 0.0305	0.1817
*atpF*_CDS	0.0142±0.0058	0.0497±0.0205	0.2857	0.0238±0.0076	0.0673±0.0242	0.354	0.0285±0.0083	0.0510±0.0213	0.559
*atpH*_CDS	0	0.0178±0.0179	0	0	0.0969±0.0443	0	0	0.0776±0.0394	0
*atpI*_CDS	0.0054±0.0031	0.0333±0.0137	0.1627	0.0054±0.0031	0.0860±0.0227	0.063	0.0036±0.0026	0.0982±0.0245	0.0368
*ccsA*_CDS	0.0173±0.0048	0.0643±0.0189	0.2688	0.0304±0.0064	0.1359±0.0288	0.2235	0.0317±0.0066	0.1314±0.0286	0.2412
*cemA*_CDS	0.0181±0.0061	0.0277±0.0125	0.6533	0.0500±0.0103	0.0897±0.0230	0.5575	0.0457±0.0098	0.0958±0.0240	0.4776
*clpP*_CDS	0.0063±0.0036	0.0136±0.0096	0.4643	0.0255±0.0074	0.0789±0.0242	0.3228	0.0233±0.0071	0.0636±0.0215	0.3668
*infA*_CDS	0	0.0227±0.0228	0	0.0233±0.0136	0.0480±0.0343	0.4859	0.0233±0.0135	0.0742±0.0438	0.3136
*matK*_CDS	0.0190±0.0040	0.0526±0.0138	0.3617	0.0609±0.0073	0.1482±0.0246	0.4105	0.0587±0.0071	0.1535±0.0249	0.3821
*ndhA*_CDS	0.0097±0.0034	0.0479±0.0140	0.2029	0.0242±0.0055	0.1709±0.0283	0.1416	0.0210±0.0051	0.1551±0.0268	0.1355
*ndhB*_CDS	0.0009±0.0009	0.0025±0.0025	0.361	0.0036±0.0018	0.0099±0.0050	0.3602	0.0027±0.0015	0.0074±0.0043	0.3589
*ndhC*_CDS	0.0035±0.0035	0.0267±0.0191	0.1324	0.0160±0.0075	0.0774±0.0336	0.2063	0.0124±0.0066	0.0772±0.0335	0.1605
*ndhD*_CDS	0.0096±0.0029	0.0324±0.0098	0.2969	0.0226±0.0045	0.1158±0.0195	0.1953	0.0180±0.0040	0.1083±0.0190	0.1664
*ndhE*_CDS	0.0042±0.0042	0.0684±0.0348	0.0607	0.0082±0.0058	0.1794±0.0637	0.0457	0.0165±0.0083	0.1820±0.0653	0.0906
*ndhF*_CDS	0.0230±0.0036	0.0470±0.0104	0.489	0.0505±0.0055	0.1666±0.0216	0.3032	0.0417±0.0050	0.1543±0.0205	0.2704
*ndhG*_CDS	0.0124±0.0055	0.0342±0.0173	0.3615	0.0379±0.0098	0.2063±0.0498	0.1836	0.0252±0.0080	0.1760±0.0434	0.1433

*ndhH*_CDS	0.0044±0.0022	0.0667±0.0164	0.0659	0.0204±0.0048	0.2092±0.0328	0.0977	0.0228±0.0051	0.1797±0.0293	0.1266
*ndhI*_CDS	0	0.0513±0.0232	0	0.0123±0.0055	0.1736±0.0475	0.0706	0.0122±0.0055	0.1409±0.0434	0.0865
*ndhJ*_CDS	0.0056±0.0040	0.0267±0.0155	0.2094	0.0056±0.0040	0.0357±0.0180	0.1565	0.0112±0.0056	0.0448±0.0203	0.2505
*ndhK*_CDS	0.0071±0.0035	0.0059±0.0059	1.1964	0.0242±0.0066	0.0963±0.0249	0.2517	0.0252±0.0068	0.0993±0.0253	0.2536
*petA*_CDS	0	0.0045±0.0045	0	0.0137±0.0044	0.1004±0.0225	0.1367	0.0137±0.0044	0.1054±0.0230	0.1303
*petB*_CDS	0	0.0062±0.0062	0	0	0.0871±0.0251	0	0	0.0951±0.0265	0
*petD*_CDS	0.0057±0.0040	0.0159±0.0113	0.3585	0.0028±0.0028	0.1736±0.0424	0.0163	0.0028±0.0028	0.1554±0.0399	0.0181
*petG*_CDS	0	0.0309±0.0310	0	0.0126±0.0126	0.1418±0.0728	0.0889	0.0128±0.0129	0.0987±0.0580	0.1301
*petL*_CDS	0.0145±0.0146	0.0919±0.0665	0.1579	0.0446±0.0260	0.0435±0.0438	1.0263	0.0294±0.0209	0.1418±0.0861	0.2076
*petN*_CDS	0	0	/	0	0.0397±0.0399	0	0	0.0397±0.0399	0
*psaA*_CDS	0.0006±0.0006	0.0241±0.0067	0.0244	0.0012±0.0008	0.0917±0.0137	0.0128	0.0006±0.0006	0.0903±0.0137	0.0065
*psaB*_CDS	0.0012±0.0009	0.0112±0.0046	0.1076	0.0024±0.0012	0.0817±0.0131	0.0295	0.0012±0.0009	0.0773±0.0127	0.0156
*psaC*_CDS	0	0.0577±0.0337	0	0	0.2155±0.0740	0	0	0.1945±0.0695	0
*psaI*_CDS	0.0121±0.0121	0	/	0.0121±0.0122	0	/	0.0245±0.0174	0	/
*psaJ*_CDS	0	0.0720±0.0421	0	0.0115±0.0116	0.1809±0.0733	0.0638	0.0115±0.0115	0.0961±0.0492	0.1198
*psbA*_CDS	0	0.0278±0.0114	0	0	0.1041±0.0237	0	0	0.1148±0.0249	0
*psbB*_CDS	0.0017±0.0012	0.0207±0.0079	0.0819	0.0043±0.0019	0.1008±0.0185	0.0422	0.0042±0.0019	0.1125±0.0199	0.0377
*psbC*_CDS	0	0.0225±0.0080	0	0	0.0761±0.0152	0	0	0.0701±0.0145	0
*psbD*_CDS	0	0.0223±0.0092	0	0.0038±0.0022	0.0529±0.0143	0.0725	0.0038±0.0022	0.0493±0.0139	0.0776
*psbE*_CDS	0	0	/	0	0.0634±0.0324	0	0	0.0634±0.0324	0
*psbF*_CDS	0	0	/	0.0121±0.0121	0.0953±0.0561	0.1268	0.0121±0.0121	0.0953±0.0561	0.1268
*psbH*_CDS	0	0.0428±0.0307	0	0.0239±0.0120	0.1109±0.0511	0.2155	0.0239±0.012	0.0871±0.0444	0.2741
*psbI*_CDS	0	0.0648±0.0466	0	0	0.0317±0.0319	0	0	0.0324±0.0330	0
*psbJ*_CDS	0.0118±0.0118	0	/	0.0118±0.0119	0.0926±0.0550	0.1278	0	0.0947±0.0562	0
*psbK*_CDS	0	0	/	0.0224±0.0130	0.0913±0.0471	0.2459	0.0224±0.0130	0.0913±0.0471	0.2459
*psbL*_CDS	0	0	/	0	0	/	0	0	/

*psbM*_CDS	0	0	/	0	0.0367 ± 0.0369	0	0	0.0367 ± 0.0369	0
*psbN*_CDS	0	0	/	0	0.0262±0.0263	0	0	0.0262±0.0263	0
*psbT*_CDS	0.0291±0.0207	0.0354±0.0356	0.8235	0.0288±0.0205	0	/	0.0293±0.0209	0.0351±0.0353	0.8338
*psbZ*_CDS	0	0	/	0.0143±0.0102	0.0461±0.0328	0.3107	0.0143±0.0102	0.0461±0.0328	0.3107
*rbcL*_CDS	0.0009±0.0009	0.0209±0.0079	0.0441	0.0018±0.0013	0.1013±0.0184	0.0182	0.0028±0.0016	0.0783±0.0160	0.0353
*rpl2*_CDS	0.0032±0.0023	0.0099±0.0070	0.3264	0.0032±0.0023	0.0099±0.0070	0.3249	0.0032±0.0023	0.0099±0.0070	0.3243
*rpl14*_CDS	0	0.0363±0.0212	0	0.0108±0.0063	0.0863±0.0334	0.1252	0.0108±0.0062	0.0871±0.0336	0.1236
*rpl16*_CDS	0	0.0513±0.0260	0	0.0031±0.0031	0.1664±0.0505	0.0186	0.0031±0.0031	0.2003±0.0575	0.0155
*rpl20*_CDS	0.0064±0.0046	0.0260±0.0185	0.2475	0.0195±0.0080	0.1138±0.0420	0.171	0.0261±0.0093	0.0835±0.0353	0.3122
*rpl22*_CDS	0.0087±0.0050	0.0614±0.0254	0.1413	0.0188±0.0074	0.1696±0.0478	0.1107	0.0277±0.0091	0.1168±0.0377	0.2372
*rpl32*_CDS	0.0227±0.0132	0.0303±0.0304	0.7494	0.0303±0.0153	0.0307±0.0309	0.9875	0.0075±0.0075	0	/
*rpl33*_CDS	0.0068±0.0068	0	/	0.0068±0.0069	0.0836±0.0430	0.0819	0	0.0834±0.0429	0
*rpl36*_CDS	0	0	/	0	0	/	0	0	/
*rpoA*_CDS	0.0025±0.0018	0.0344±0.0132	0.0731	0.0360±0.0069	0.0908±0.0219	0.397	0.0339±0.0066	0.1064±0.0241	0.3186
*rpoB*_CDS	0.0069±0.0017	0.0265±0.0061	0.2605	0.0167±0.0026	0.0891±0.0117	0.1877	0.0148±0.0025	0.0890±0.0117	0.1667
*rpoC1*_CDS	0.0040±0.0016	0.0297±0.0088	0.1342	0.0124±0.0028	0.0900±0.0162	0.1374	0.0152±0.0031	0.1119±0.0182	0.1354
*rpoC2*_CDS	0.0077±0.0016	0.0292±0.0059	0.2627	0.0360±0.0034	0.1207±0.0128	0.2982	0.0337±0.0033	0.1107±0.0122	0.3042
*rps2*_CDS	0.0035±0.0025	0.0431±0.0178	0.0823	0.0143±0.0051	0.1231±0.0321	0.1162	0.0143±0.0051	0.1224±0.0319	0.117
*rps3*_CDS	0	0.0301±0.0152	0	0.0155±0.0055	0.1344±0.0347	0.115	0.0155±0.0055	0.1510±0.0371	0.1028
*rps4*_CDS	0.0066±0.0038	0.0149±0.0106	0.4418	0.0110±0.0049	0.0862±0.0265	0.1275	0.0088±0.0044	0.0699±0.0236	0.1256
*rps7*_CDS	0	0.0205±0.0145	0	0	0.0316±0.0184	0	0	0.0529±0.0242	0
*rps8*_CDS	0.0066±0.0047	0.0101±0.0101	0.6554	0.0252±0.0092	0.1159±0.0368	0.2171	0.0183±0.0078	0.1048±0.0348	0.1747
*rps11*_CDS	0.0033±0.0033	0.0288±0.0167	0.1133	0.0098±0.0057	0.1823±0.0463	0.0538	0.0065±0.0046	0.1969±0.0493	0.0331
*rps12*_CDS	0.0037±0.0037	0.0102±0.0102	0.3635	0.0037±0.0037	0.0312±0.0182	0.1187	0	0.0206±0.0146	0
*rps14*_CDS	0.0131±0.0076	0	/	0.0221±0.0099	0.0593±0.0300	0.3725	0.0265±0.0109	0.0602±0.0304	0.4399
*rps15*_CDS	0.0103±0.0073	0.0380±0.0271	0.2718	0.0634±0.0186	0.2925±0.0946	0.2168	0.0640±0.0187	0.2800±0.0898	0.2285

*rps16*_CDS	0.0138±0.0080	0	/	0.0519±0.0158	0.1261±0.0599	0.4114	0.0472±0.0151	0.1236±0.0586	0.3819
*rps18*_CDS	0	0.0395±0.0230	0	0.0089±0.0063	0.0677±0.0308	0.1319	0.0089±0.0063	0.0397±0.0231	0.2249
*rps19*_CDS	0.0095±0.0067	0.0670±0.0343	0.1411	0.0141±0.0081	0.1458±0.0534	0.0965	0.0142±0.0082	0.1618±0.0559	0.0876
*ycf1*_CDS	0.0076±0.0013	0.0047±0.0021	1.6043	0.0174±0.0020	0.0237±0.0048	0.7317	0.0165±0.0019	0.0234±0.0047	0.7072
*ycf2*_CDS	0.0112±0.0015	0.0079±0.0024	1.4247	0.0193±0.0020	0.0201±0.0039	0.9582	0.0150±0.0018	0.0150±0.0033	0.9992
*ycf3*_CDS	0.0065±0.0046	0.0513±0.0259	0.1267	0.0032±0.0032	0.1369±0.0447	0.0236	0.0032±0.0032	0.1241±0.0429	0.026
*ycf4*_CDS	0.0049±0.0034	0	/	0.0122±0.0055	0.0445±0.0183	0.2745	0.0073±0.0042	0.0443±0.0182	0.1652

Note: FD: *F. dibotrys*; FE: *F. esculentum* subsp. *ancestrale*; FT: *F. tataricum*; FL: *F. luojishanense*

Table S3 The primers designed for divergence hotspot regions amplification

**Primer**		**Sequence**
ycf2-trnL	F	GGATTTTCCCGGATGAACTGA
	R	TAAAGAGCGTGGAGGTTCGA
psbE-petL	F	GTGCTTCCAGACATGCTCAG
	R	AGAGTAGGCATGAAGGAGCT
psbM-trnD	F	ACGTCACAATAACTCGGGATT
	R	TGTAGTTCAATTGGTCAGAGCAC
ycf3-trnS	F	CGTGATCTAGGCATAGGTACCA
	R	GCCACCTCTCCTACACAACA
rps4-trnT	F	GGTAACGCGACATAAAGACTCC
	R	CCGCTTAGCTCAGAGGTTAGA
psbI-trnS	F	TGCTTACTCTCAAACTCTTCGT
	R	AGATGGCTGAGTGGACGAAA
ndhF-rpl32	F	CATTGGCTCTTACCTCTTTCGA
	R	GCCGAATATCCTTTGCTTTTCC
rps16-trnQ	F	ATCCCTCCAATTTTGAGCCG
	R	TTCGAATCCTTCCGTCCCAG
atpF	F	CCCTTCCCGAACCAAACTTG
	R	CCGTTTGAAGTTCAGGCACA
rpoC2	F	CTTGTGTCCGAAATTCCCCG
	R	TACCGCGATCTTCTGCCATA
petN-psbM	F	GGGACAATGCGTAACAACAGA
	R	AAGCAGGCTAGAATCAACAGT
trnS-trnG	F	ACTCAGCCATCTCTCCCTATTG
	R	ACCACTAAACTATACCCGCTCC
trnE-trnT	F	AACCACTAGACGATGAGGGC
	R	CCGATGACTTACGCCTTACC
psaA-ycf3	F	TTACTTCTGGTTCCGGCGAA
	R	TTGGTTGAAGATCACGAGGC

Table S4 List of plastome sequences included in the phylogenetic analyses

No.	Family	Taxon	Accesion number
1	Caryophyllaceae	*Colobanthus quitensis*	NC_028080.1
2	Caryophyllaceae	*Dianthus longicalyx*	KM668208.1
3	Caryophyllaceae	*Lychnis wilfordii*	NC_035225.1
4	Caryophyllaceae	*Agrostemma githago*	NC_023357.1
5	Caryophyllaceae	*Silene latifolia* subsp. *alba*	KT962040.1
6	Caryophyllaceae	*Silene chalcedonica*	NC_023359.1
7	Caryophyllaceae	*Silene paradoxa*	NC_023360.1
8	Caryophyllaceae	*Silene conoidea*	NC_023358.1
9	Caryophyllaceae	*Silene latifolia*	NC_016730.1
10	Caryophyllaceae	*Silene conica*	NC_016729.1
11	Caryophyllaceae	*Silene noctiflora*	NC_016728.1
12	Caryophyllaceae	*Silene capitata*	NC_035226.1
13	Caryophyllaceae	*Silene vulgaris*	NC_016727.1
14	Polygonaceae	*Fagopyrum tataricum* cultivar Miqiao 1	KX085498.1
15	Polygonaceae	*Fagopyrum tataricum*	NC_027161.1
16	Polygonaceae	*Fagopyrum esculentum* subsp. *ancestrale*	EU254477.1
17	Polygonaceae	*Rheum palmatum*	NC_027728.1
18	Polygonaceae	*Rheum wittrockii*	NC_035950.1
19	Polygonaceae	*Oxyria sinensis*	NC_032031.1
20	Polygonaceae	*Fagopyrum cymosum*	KP404630.1
21	Polygonaceae	*Fagopyrum dibotrys*	KY275181
22	Polygonaceae	*Fagopyrum luojishanense*	KY275182
23	Chenopodiaceae	*Beta vulgaris*	EF534108.1
24	Chenopodiaceae	*Beta vulgaris* subsp. *vulgaris*	KR230391.1
25	Chenopodiaceae	*Spinacia oleracea*	AJ400848.1
26	Chenopodiaceae	*Chenopodium album*	NC_034950.1
27	Chenopodiaceae	*Chenopodium quinoa* voucher IT123455	NC_034949.1
28	Chenopodiaceae	*Chenopodium quinoa*	KY635884.1
29	Chenopodiaceae	*Bienertia sinuspersici*	KU726550.2
30	Chenopodiaceae	*Salicornia bigelovii*	NC_027226.1
31	Chenopodiaceae	*Salicornia europaea*	NC_027225.1
32	Chenopodiaceae	*Salicornia brachiata*	NC_027224.1
33	Chenopodiaceae	*Haloxylon persicum*	NC_027669.1
34	Chenopodiaceae	*Haloxylon ammodendron*	NC_027668.1
35	Amaranthaceae	*Amaranthus hypochondriacus*	NC_030770.1
36	Amaranthaceae	*Amaranthus tricolor*	KX094399.1
37	Cactineae/Cactaceae	*Carnegiea gigantea*	NC_027618.1
38	Cactineae/Montiaceae	*Cistanthe longiscapa*	NC_035140.1
39	Droseraceae	*Drosera rotundifolia*	NC_029770.1
40	Droseraceae	*Dionaea muscipula*	NC_035417.1
41	Droseraceae	*Aldrovanda vesiculosa*	NC_035416.1
42	Droseraceae	*Drosera regia*	NC_035415.1

43	Aizoaceae	*Mesembryanthemum crystallinum*	NC_029049.1
44	Outgroup	*Osyris alba*	NC_027960
45	Outgroup	*Champereia manillana*	NC_034931

Table S5 Model in ML and BI analysis based on different datasets

	Best-fit model in ML (ModelFinder)	Best-fit model In BI (Modeltest)
Complete cp genome	GTR+F+R3	TVM+G
50 shared genes	GTR+F+R4	GTR+I+G

Table S6 List of the 50 genes used in the phylogenetic analyses

No.	Gene	No.	Gene
1	*atpA*	26	*psbJ*
2	*atpB*	27	*psbK*
3	*atpE*	28	*psbM*
4	*atpF*	29	*psbN*
5	*atpH*	30	*psbT*
6	*atpI*	31	*psbZ*
7	*ccsA*	32	*rbcL*
8	*cemA*	33	*rpl2*
9	*petA*	34	*rpl14*
10	*petG*	35	*rpl16*
11	*petL*	36	*rpl20*
12	*petN*	37	*rpl22*
13	*psaA*	38	*rpoA*
14	*psaB*	39	*rpoB*
15	*psaC*	40	*rpoC1*
16	*psaI*	41	*rps2*
17	*psaJ*	42	*rps3*
18	*psbA*	43	*rps4*
19	*psbB*	44	*rps7*
20	*psbC*	45	*rps8*
21	*psbD*	46	*rps11*
22	*psbE*	47	*rps12*
23	*psbF*	48	*rps14*
24	*psbH*	49	*rps15*
25	*psbI*	50	*ycf4*

Supplementary figures

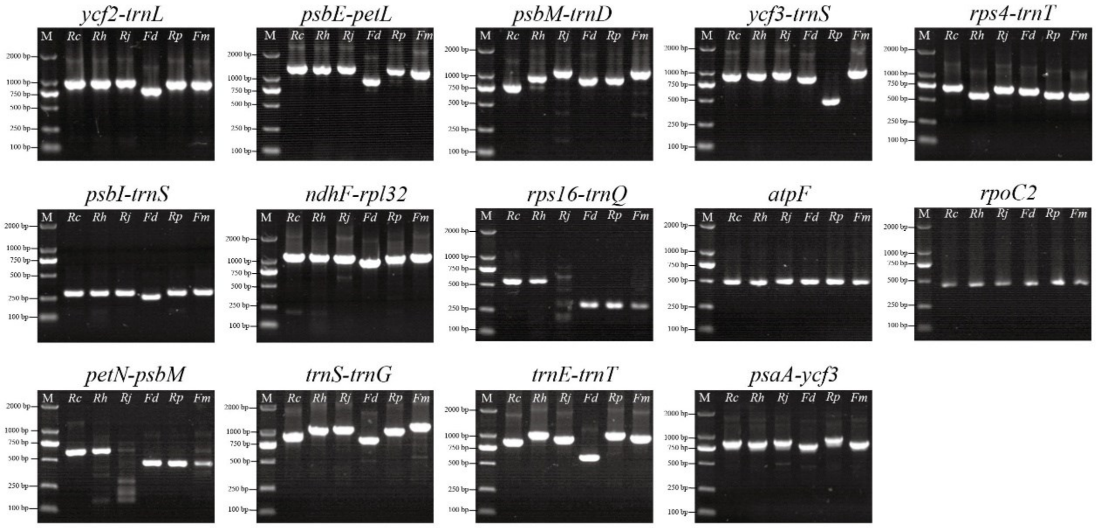

Fig. S1 Validation of 14 molecular markers derived from hotspot regions in cp genomes. 1.5% agarose gels profiles of PCR fragments from six Polygonaceae species using 14 primer pairs. Names at the top of each figure represent different hotspot regions. Abbreviated species names are shown on the top of each lanes: *Rc*: *Rumex crispus, Rh*: *Rheum hotaoense, Rj*: *Reynoutria japonica, Fd*: *Fagopyrum dibotrys Rp*: *Rheum palmatum*, *Fm*: *Fallopia multiflora,* M: DL2000 DNA ladder.

Supplementary datasets

Supplementary Dataset 1 is provided in extra xls file.

Supplementary Dataset 1 Sanger sequencing of results for six Polygonaceae species based on 14 molecular markers derived from hotspot regions.
